# Radiofrequency Ablation for Internal Hemorrhoids: A Case Series

**DOI:** 10.7759/cureus.61405

**Published:** 2024-05-31

**Authors:** Kean leong Koay, Nabil Mohammad Azmi, Soma Chandrakanthan, Diana Melissa Dualim, Nur Afdzillah Abdul Rahman

**Affiliations:** 1 Department of Surgery, Faculty of Medicine, The National University of Malaysia, Kuala Lumpur, MYS

**Keywords:** colorectal, surgery, haemorrhoids, laser, gastrointestinal bleeding

## Abstract

Internal hemorrhoids are a common issue in general surgery and are one of the leading causes of lower gastrointestinal bleeding globally. Numerous treatment options exist for managing this challenging condition. One relatively new treatment method is radiofrequency ablation for internal hemorrhoids (RAFAELO). According to the limited publications, this method is described as simple, quick, and safe. In this case series, we present five patients with internal hemorrhoids who were treated using the RAFAELO method and discuss their management and outcomes.

## Introduction

Internal hemorrhoids, defined as the engorgement of anal cushions, are among the most common causes of lower gastrointestinal bleeding worldwide [1]. Although benign, this condition can significantly impact a patient's quality of life, causing symptoms such as rectal bleeding, fecal soiling, and pruritus ani [1]. Numerous treatment options exist for hemorrhoids, including rubber band ligation, stapler hemorrhoidopexy, hemorrhoidal arterial ligation, and open hemorrhoidectomy [1]. Recently, a new technique called radiofrequency ablation for internal hemorrhoids (RAFAELO) has been introduced, offering a safe and quick alternative for treating this condition [2]. In this case series, we present five patients with internal hemorrhoids who underwent RAFAELO, and we compare their outcomes with those reported in other studies.

## Case presentation

Case 1

**Figure 1:**
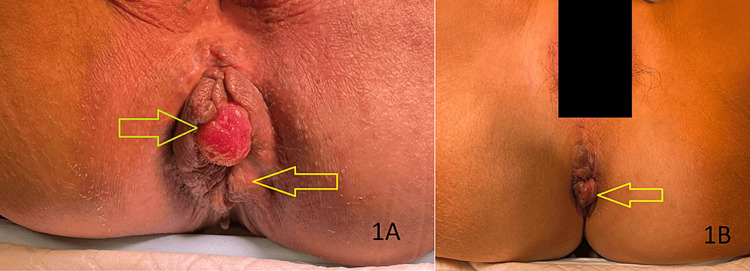
Intraoperative pictures of Case 1 (A) Preoperative photo of Case 1, showing grade 3 internal hemorrhoids at 11 o'clock (green arrow) and 5 o'clock (yellow arrow). (B) Postoperative photo of Case 1, showing residual hemorrhoids at 5 o'clock (yellow arrow), while that at 11 o'clock is no longer visible.

SSC, a 71-year-old Chinese woman, has experienced intermittent painless fresh rectal bleeding for the past two years. She reported no mucus in her stool, tenesmus, weight loss, loss of appetite, constipation, or changes in bowel habits. An abdominal examination revealed no abnormalities, and perineal examination along with proctoscopy identified grade III internal hemorrhoids at the 11 and 5 o'clock positions, without any signs of recent bleeding. A digital rectal examination found no additional masses inside or outside the rectum. Following her initial visit, a colonoscopy was performed, which yielded unremarkable results. The patient underwent the RAFAELO procedure on August 14, 2023, under spinal anesthesia, with intraoperative findings shown in Figure 1.

Postoperatively, she experienced no pain, even after the spinal anesthesia wore off. At the two-week follow-up, she noted that her hemorrhoids persisted but had significantly reduced in size. She had postoperative bleeding, which was observed only during the two-week clinic review, and a sigmoidoscopy revealed a bleeding internal hemorrhoid, which was successfully treated with rubber band ligation.

By six weeks postoperation, there were no signs of recurrent bleeding. Although she reported a small persistence of hemorrhoids, they had improved compared to her preoperative condition. She did not experience any other surgery-related complications and expressed overall satisfaction with the procedure.

Case 2

**Figure 2:**
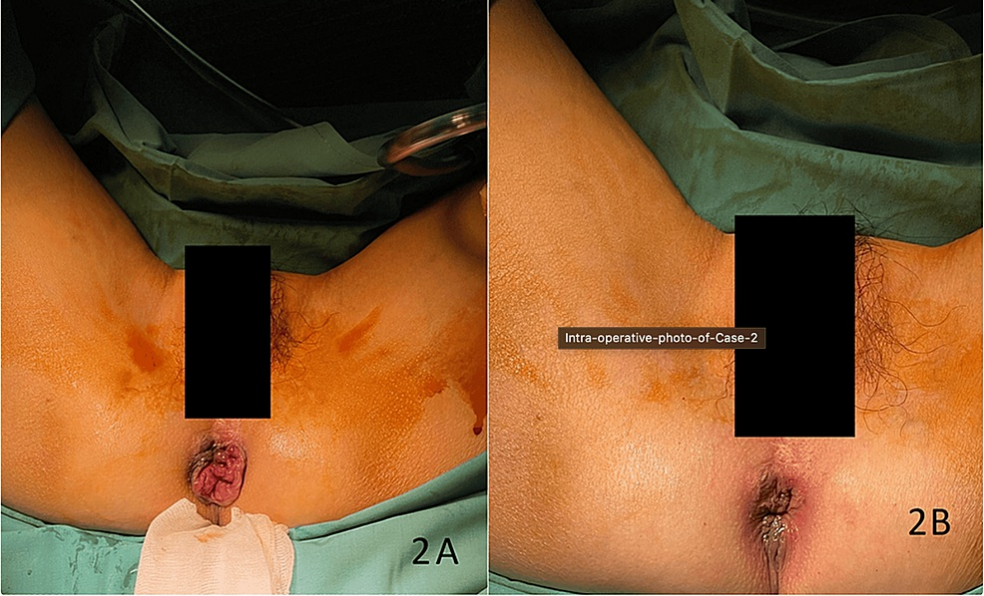
Intraoperative photo of Case 2 (A) Preoperative photo of Case 2, showing grade III internal hemorrhoids at 3, 7, and 11 o'clock. (B) Postoperative photo of Case 2, showing marked reduction in the size of hemorrhoids.

AW, a 56-year-old woman, initially presented to our clinic with fresh rectal bleeding that had persisted for the past year. She reported no perianal pain, tenesmus, mucus in her stool, or constitutional symptoms, and her bowel habits remained unchanged. Examination revealed grade III internal hemorrhoids at the 3, 7, and 11 o'clock positions, with no palpable mass detected during the digital rectal examination. A preoperative colonoscopy yielded normal results. Madam AW underwent the RAFAELO under spinal anesthesia, with intraoperative findings illustrated in Figure 2.

Following the procedure, she experienced mild pain at the operative site, with a pain score of 2, which did not require analgesia. By the two-week mark, her pain had completely resolved, and although her hemorrhoids persisted, they had reduced in size. She did not encounter any postoperative complications, including bleeding or infection. Overall, she expressed satisfaction with the surgery.

Case 3

**Figure 3:**
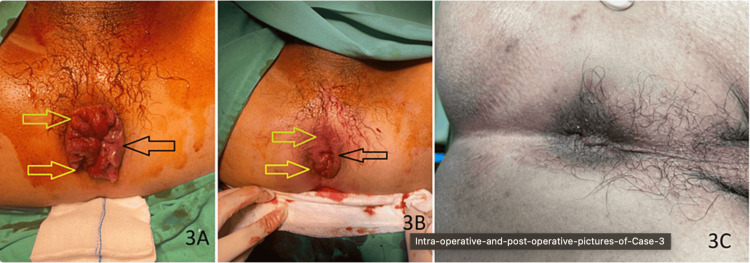
Intraoperative and postoperative pictures of Case 3 (A) Preoperative photo of Case 3, showing grade III hemorrhoids at 3 (black arrow), 7 (yellow arrow), and 11 (green arrow) o'clock respectively. (B) Postoperative photo of Case 3, showing a slight reduction in the size of hemorrhoids. (C) Photo of Case 3 at post-op two weeks, showing that hemorrhoids are almost completely resolved.

CCO, a 49-year-old man, presented with a protruding rectal mass that had persisted for two months. He reported no rectal bleeding, pain, or mucus in his stool and noted no changes in bowel habits. An abdominal examination revealed no palpable mass, while a perineal examination and proctoscopy identified grade III internal hemorrhoids at the 3, 7, and 11 o'clock positions. A colonoscopy detected only a small diverticulum in the sigmoid colon, with no other concerning masses or lesions. CCO underwent the RAFAELO under spinal anesthesia, with intraoperative findings depicted in Figure 3.

Following the procedure, he experienced mild sharp pain with a pain score of 1, which did not require any pain medication. At the two-week postoperative check-up, he reported that his prolapsed hemorrhoids had completely resolved, with no hemorrhoids detected during the perineal examination (Figure 3, Panel C). His recovery was smooth, with no postoperative complications.

At the six-week postoperative review, the perineal inspection showed no protruding hemorrhoids, although proctoscopy revealed a small recurrence at the 3 and 11 o'clock positions. He did not have any bleeding or infection after the surgery and was overall satisfied with the outcome.

Case 4

**Figure 4:**
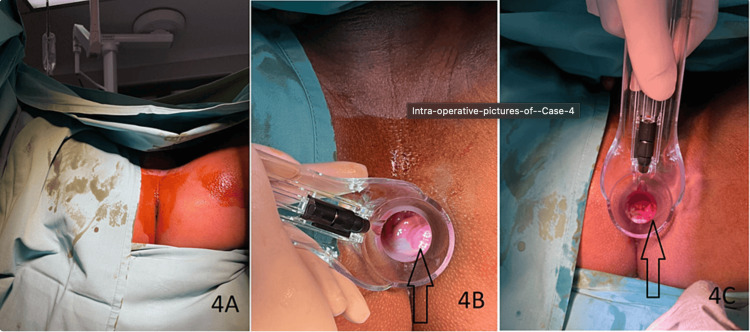
Intraoperative pictures of Case 4 (A) Preoperative photo of case 4 showing that no hemorrhoids were visible externally. (B) Intraoperative proctoscopy photo showing small grade II internal hemorrhoids visible at 5 o'clock (black arrow). (C) Post-RAFAELO proctoscopy photo showing a smaller hemorrhoidal cushion at 5 o'clock (black arrow).

KA, a 34-year-old man, initially presented to the emergency department with painless rectal bleeding that had persisted for six months before being referred to our outpatient department. Upon further inquiry, he described the bleeding as painless and not accompanied by any changes in bowel habits. Abdominal examination revealed no significant findings, while a perineal examination identified a grade II internal hemorrhoid at the 5 o'clock position. A preoperative sigmoidoscopy confirmed the diagnosis of internal hemorrhoids, with no tumors or suspicious lesions observed up to the sigmoid colon. KA underwent the RAFAELO under general anesthesia, with intraoperative findings depicted in Figure 4.

After the procedure, the patient reported no pain and encountered neither immediate nor delayed postoperative complications. During the two-week clinic review, his hemorrhoids remained present, although the rectal bleeding had ceased. At six weeks postoperatively, his small internal hemorrhoids persisted, but he noted that the rectal bleeding had stopped. He did not experience any additional postoperative complications, and when asked about his satisfaction with the surgery, his response was ambivalent.

Case 5

**Figure 5:**
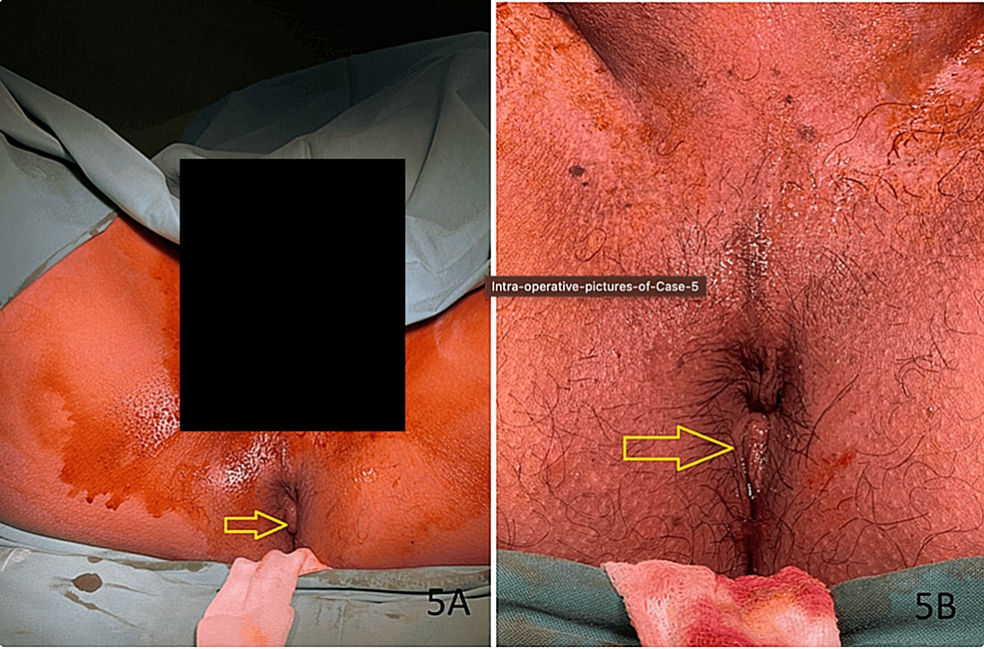
Intraoperative pictures of Case 5 (A) Preoperative photo of Case 5 showing hemorrhoid cushion at 7 o'clock (yellow arrow). (B) Postoperative photo showing a reduction in the size of the hemorrhoidal cushion.

Mr. MS, a 35-year-old man, visited our outpatient department with fresh rectal bleeding as his primary concern. He denied experiencing perineal pain, tenesmus, mucus in stool, weight loss, or loss of appetite. Upon examination, a grade II internal hemorrhoid was observed at the 7 o'clock position, and digital examination did not reveal any palpable mass in the rectum. A preoperative sigmoidoscopy showed a grade 1 internal hemorrhoid without evidence of recent bleeding. He underwent the RAFAELO procedure on August 14, 2023, under general anesthesia. The intraoperative findings are depicted in Figure 5.

After the procedure, the patient reported no pain in the perineal region, and he did not experience any immediate or delayed postoperative complications. At the two-week follow-up, he did not report any rectal bleeding, and a proctoscopy examination revealed only a small residual hemorrhoid cushion. However, at six weeks postoperatively, the hemorrhoid persisted without resolution. Despite the absence of other postoperative complications, the patient expressed dissatisfaction with the surgery due to the persistence of his hemorrhoids.

## Discussion

Hemorrhoids remain a prevalent cause of lower gastrointestinal bleeding worldwide, estimated to affect half the population by the age of 50 [1]. They occur due to the engorgement and prolapse of the anal cushion along with the hemorrhoidal plexus. Although their etiology is multifactorial, several factors are commonly associated with hemorrhoidal disease, including obesity, increased intraabdominal pressure, constipation, straining during defecation, low fiber intake, weakened pelvic floor muscles, anal sphincter injury, and pregnancy [3].

Hemorrhoids are classified into internal and external types based on their location relative to the dentate line [1]. External hemorrhoids arise below the dentate line and are supplied by somatic nerves, while internal hemorrhoids originate above the dentate line and are supplied by visceral nerve fibers [1]. Consequently, irritation of external hemorrhoids causes intense pain, whereas internal hemorrhoids are typically painless. Another classification system described by Goligher categorizes hemorrhoids based on their prolapse and reducibility: Grade 1 hemorrhoids do not prolapse, grade 2 hemorrhoids prolapse but are spontaneously reducible, grade 3 hemorrhoids require manual reduction, and grade 4 hemorrhoids are irreducible (Table 1) [4].

**Table 1:** Goligher classification of internal hemorrhoids

Grade	Description
1	Normal appearance externally, bleeding but not prolapsing
2	Anal cushions prolapse on straining but recovers spontaneously
3	Anal cushions prolapse on straining but requires manual reduction
4	Permanent prolapse and irreducible

Patients with hemorrhoids often experience periodic rectal bleeding, pruritus, and fecal soiling [5]. Although these symptoms may seem minor, they can significantly impact their quality of life. However, many sufferers avoid seeking medical treatment due to fear of pain during treatment and the intimate nature of the condition [6].

Treatment options for hemorrhoidal disease can be broadly categorized into non-excision and excision methods [1]. Non-excision methods encompass techniques such as rubber band ligation, sclerotherapy, cryotherapy, laser hemorrhoidoplasty, hemorrhoidal artery ligation, and radiofrequency ablation. Excision methods include classic Milligan-Morgan or Ferguson hemorrhoidectomy and stapler hemorrhoidopexy. For grade 1-2 hemorrhoids, rubber band ligation is often the initial procedure of choice, which can be performed in an outpatient setting without anesthesia. Compared to sclerotherapy, rubber band ligation typically yields better outcomes, although reported recurrence rates vary widely, ranging from 11% to 50% [7]. Hemorrhoidal artery ligation is a newer technique that involves using a modified proctoscope with a Doppler probe to localize hemorrhoidal arteries, which are then ligated manually [7]. For grade 3-4 hemorrhoids, excisional surgeries like Milligan-Morgan or Ferguson hemorrhoidectomy and stapler hemorrhoidopexy demonstrate high success rates. However, these procedures are often associated with prolonged post-operative pain and recovery times [2]. Additionally, excisional procedures carry the risk of post-operative stricture and incontinence. Although these complications are rare, they can be debilitating and challenging to manage [8].

A study published in 2012 compared 2840 cases of open hemorrhoidectomy and examined the incidence of complications associated with these procedures [9]. Severe bleeding occurred in approximately 1.9% of patients undergoing open hemorrhoidectomy, stenosis in 1.9%, and anal hypotonia in 0.4% of these patients [9]. Regarding recurrence, open surgery is considered the gold standard with a recurrence rate as low as 1%, whereas stapler hemorrhoidectomy has a higher recurrence rate of 7% [10]. However, open hemorrhoidectomy is associated with a higher risk of immediate and early postoperative complications including bleeding, surgical site infection, and readmission within 30 days postoperatively [11].

RAFAELO is a relatively novel procedure that offers potential advantages such as reduced procedural time and post-operative pain compared to other existing modalities [7]. In RAFAELO, radiofrequency waves are converted into thermal energy, causing coagulation necrosis of the tissues in contact by inducing ionic agitation and frictional heating [7]. Although radiofrequency ablation (RFA) has been widely used in other medical areas such as varicose veins and Barrett’s esophagus, literature on its application for hemorrhoids is currently limited [7]. RAFAELO presents several advantages over other modalities, including its suitability for outpatient settings under local anesthesia, applicability for hemorrhoids up to grade 3, and a notably lower recurrence rate compared to rubber band ligation and hemorrhoidal artery ligation [5]. In our case series, each patient had to spend an additional Malaysian Ringgit 788.00 (USD 170.00) in purchasing the radiofrequency probe and renting the radiofrequency generator from a medical company.

There were notable publications regarding RAFAELO. The first study that is relevant to the discussion was conducted in Cologne, Germany, and published in 2022. It was a prospective two-center study aiming to evaluate the suitability of the RAFAELO procedure for outpatient settings [5]. This study involved 98 patients with grade 3 hemorrhoids, all of whom underwent the RAFAELO procedure on an outpatient basis under local anesthesia. The study reported a low recurrence rate (13.7%) at two years, along with reduced post-operative pain. Approximately 30% of patients reported a pain score of 0, and 60.2% of patients did not require analgesia [5]. Moreover, the study reported a low complication rate, with 11.2% experiencing minor complications and only 8.2% experiencing major complications that required antibiotics, secondary intervention, or hospitalization [5].

Another case series conducted in the United Kingdom studied 42 patients undergoing RAFAELO, including those with grade 1-3 hemorrhoids [2]. The findings of this study were consistent with the previous study in Germany, with up to 38% of patients reporting a pain score of 0 immediately post-operatively and at one-month follow-up. Only 33% of patients required analgesia, with a median postoperative pain score of 2.5/10 [2]. Most patients expressed satisfaction with the procedure, with a median satisfaction score of 8.5/10 [2].

In our study, there were no cases of immediate post-operative complications, including bleeding or post-operative pain. One out of five cases (20%) experienced early post-operative bleeding, noted at two weeks post-operatively, which resolved with sigmoidoscopy and rubber band ligation. The median post-operative pain score was 0, with the highest reported pain score of 2 in one subject, which resolved with an oral analgesia prescription. None of our patients experienced post-operative sepsis or pelvic infection. As demonstrated in the comparison of pre- and post-operative photos, all subjects experienced a reduction in hemorrhoid size post-operatively. While all subjects experienced persistent hemorrhoids on follow-up, three out of five subjects noted significant improvement in hemorrhoidal size, and one other subject noted improvement in symptoms. However, one subject reported no reduction in symptoms or size of hemorrhoid, possibly due to technical factors such as inadequate radiofrequency probe withdrawal time. Overall, three out of these five subjects (60%) were satisfied with their RAFAELO treatment, one was dissatisfied, and the other remained undecided regarding satisfaction with the surgery. None of our subjects experienced major complications or required readmission to the hospital.

## Conclusions

In summary, our case series demonstrates that RAFAELO is a straightforward and safe procedure for individuals suffering from internal hemorrhoids. We observed minimal postoperative complications, both minor and major, and very low levels of postoperative discomfort. While most patients still had persistent hemorrhoids after the procedure, the majority experienced a significant reduction in hemorrhoidal size and improvement in symptoms, leading to satisfaction with their treatment. Moreover, our case series highlights the feasibility of performing RAFAELO in a daycare setting. Based on our findings, we advocate for RAFAELO as a swift and uncomplicated treatment option for internal hemorrhoids. Longer follow-up, however, is needed to ascertain long-term outcomes such as hemorrhoid recurrence.
